# Are women cryopreserving enough oocytes for fertility preservation? A probabilistic model integrating AI-based oocyte morphology and age-dependent euploidy

**DOI:** 10.1093/hropen/hoag090

**Published:** 2026-10-14

**Authors:** Daniela P A F Braga, Amanda S Setti, Maite Del Collado, Jullin Fjeldstad, Parisa Mojiri, Melissa Cavagnoli, Edson Borges

**Affiliations:** Centro de Estudos em Reprodução Assistida, Instituto Sapientiae, São Paulo, Brazil; Equinvitro Research and Development in Reproduction, Botucatu, Brazil; Centro de Estudos em Reprodução Assistida, Instituto Sapientiae, São Paulo, Brazil; Centro de Estudos em Reprodução Assistida, Instituto Sapientiae, São Paulo, Brazil; Future Fertility, Toronto, Canada; Future Fertility, Toronto, Canada; Clínica Hope, São Paulo, Brazil; Centro de Estudos em Reprodução Assistida, Instituto Sapientiae, São Paulo, Brazil; Fertility/FERTGROUP—Medicina Reprodutiva, São Paulo, Brazil

**Keywords:** fertility preservation, oocyte cryopreservation, artificial intelligence, oocyte quality, euploidy, maternal age, preimplantation genetic testing, counselling

## Abstract

**STUDY QUESTION:**

How many mature oocytes are biologically required to achieve at least three euploid blastocysts, by maternal age and AI-derived oocyte quality, and how does this estimate compare with real-world oocyte yields in elective fertility preservation?

**SUMMARY ANSWER:**

Required oocyte numbers rise steeply with advancing maternal age and decreasing AI-derived oocyte quality, and the median number cryopreserved per fertility preservation cycle falls progressively below estimated requirements, by 12- to 30-fold in women older than 40 years.

**WHAT IS KNOWN ALREADY:**

Elective oocyte cryopreservation is increasingly used to preserve future reproductive potential, but counselling typically relies on uniform age-based oocyte targets that do not account for inter-individual variability in oocyte biological competence. Although AI-based oocyte assessment has shown promise as an objective adjunct to morphological evaluation, no biologically integrated framework has been used to translate AI-derived oocyte quality into a quantitative requirement-versus-yield comparison in fertility preservation.

**STUDY DESIGN, SIZE, DURATION:**

Retrospective cohort study with probabilistic modelling of blastulation and euploidy, using three datasets from a single private university-affiliated IVF centre. Model development used 13 782 mature oocytes from 1625 autologous ICSI cycles (1020 patients, 2021–2024) and 3325 biopsied embryos with conclusive PGT-A results (857 patients). External validation was performed in 1948 oocytes from 333 ovum donation cycles in 271 donors (2020–2024). Real-world application used 1647 elective fertility preservation cycles in 1100 patients between 2012 and 2024.

**PARTICIPANTS/MATERIALS, SETTING, METHODS:**

Each oocyte was scored using a commercially available artificial-intelligence image-analysis system that assigns a 0–10 oocyte quality score. Blastulation and euploidy were modelled by logistic regression with cluster-robust standard errors. The integrated probabilistic framework computed the minimum number of mature oocytes required to obtain at least three euploid blastocysts at a probability of 90% or greater, by maternal age category and tertile of AI-derived oocyte quality score. Model-derived requirements were compared with the median number of oocytes actually cryopreserved per fertility preservation cycle.

**MAIN RESULTS AND THE ROLE OF CHANCE:**

Blastulation increased with higher AI-derived oocyte quality score (OR 1.19 per point, 95% CI 1.18–1.21; *P* < 0.001) and decreased with maternal age (OR 0.94 per year, 95% CI 0.93–0.96; *P* < 0.001); the oocyte quality effect was externally validated in donors (OR 1.11 per point, 95% CI 1.08–1.14). Euploidy declined non-linearly with age, from 68.6% (<30 years) to 19.3% (>40 years). Estimated mature-oocyte requirements ranged from 9 (95% CI 8–11) at <30 years with high AI-derived oocyte quality to 121 (104–146) at >40 years with low AI-derived oocyte quality. The median number cryopreserved per cycle decreased from 8.3 at <30 to 4.0 at >40 years and fell progressively below estimated requirements; in women older than 40 years the observed yield was 12- to 30-fold below the estimated requirement.

**LIMITATIONS, REASONS FOR CAUTION:**

Blastulation and euploidy probabilities were derived from autologous ICSI cycles in patients with infertility-related indications; extrapolation to elective fertility preservation candidates assumes that per-oocyte biological competence, conditional on age and AI-derived quality, is transportable across populations. The probabilistic framework assumes conditional independence between blastulation and euploidy. The model estimates oocyte requirements for euploid blastocyst formation but does not directly model implantation, clinical pregnancy, or live birth.

**WIDER IMPLICATIONS OF THE FINDINGS:**

The divergence between observed yields and model-derived requirements is best interpreted not as a deficiency in retrieval practice but as an individualized benchmark. Integrating AI-based oocyte assessment with age-specific euploidy probabilities may support more personalized, biologically informed counselling, expectation-setting, and care planning, particularly for women considering oocyte cryopreservation after the mid-thirties.

**FUNDING:**

This research did not receive any specific grant from public, commercial, or not-for-profit funding agencies. Institutional support was provided by the Sapientiae Institute and FertGroup Medicina Reprodutiva.

**DISCLOSURES:**

J.F. and P.M. are employees of Future Fertility, the developer of the artificial-intelligence oocyte-scoring system used in this study. The remaining authors declare no competing interests.

**TRIAL REGISTRATION NUMBER:**

N/A.

WHAT DOES THIS MEAN FOR PATIENTS? Many women choose to freeze their eggs to keep the option of having a baby later in life. A common question is how many eggs are enough. Until now, advice has mostly been based on age alone, but eggs from women of the same age can differ a lot in quality, and quality matters for the chance that an egg becomes a healthy embryo. In this study, we combined a woman’s age with an artificial-intelligence score of egg quality to estimate how many mature eggs would typically be needed to obtain three chromosomally normal embryos, a number linked to a good chance of a future pregnancy.

We found that the number of eggs required rises steeply as age increases and as egg quality falls. A woman under 30 with high-quality eggs might need around nine eggs, whereas a woman over 40 with lower-quality eggs could need many times more. When we looked at how many eggs women actually froze, we saw that many, especially those older than 35 and particularly those over 40, froze far fewer eggs than our estimates suggest they would need, sometimes only a fraction of that number in a single treatment.

For patients, this means that freezing eggs earlier, when more good-quality eggs can usually be collected, offers a real advantage, and that more than one treatment cycle may sometimes be needed. It also shows the value of personalized counselling that considers both age and egg quality, rather than a single target number for everyone. Importantly, these are estimates of the eggs needed to reach healthy embryos, not a guarantee of a baby, since pregnancy also depends on other factors. The figures come from one fertility centre and would need to be confirmed in other clinics before being applied everywhere.

## Introduction

The age at which women have their first child has risen steadily across most developed countries, driven by social, educational, and economic factors (Baldwin and Nord, 1984; Wong and Bonifácio, 2009; Adashi and Gutman, 2018; Eurostat, 2023). This demographic shift has occurred despite a well-established decline in female fertility that accelerates from the mid-thirties onward and is attributable to both a decrease in ovarian reserve and an age-related increase in meiotic errors leading to aneuploidy (Mikwar *et al.*, 2020). Elective oocyte cryopreservation has emerged as a means by which women can preserve their reproductive potential in anticipation of age-related fertility decline (Varlas *et al.*, 2021). Whether the number of oocytes currently cryopreserved in routine clinical practice is biologically sufficient to achieve a reasonable probability of future reproductive success, however, remains uncertain.

Oocyte cryopreservation is a pivotal component of assisted reproductive technology. Although the first pregnancies arising from frozen oocytes were reported in the 1980s (Chen, 1986), the method gained recognition as a secure and effective approach for achieving successful *in vitro* fertilization outcomes with the advent of vitrification (Coello *et al.*, 2018). Vitrification is now regarded as the gold standard for oocyte cryopreservation, with reproductive outcomes that approach those of fresh oocytes in experienced hands. Comparability is nonetheless dependent on oocyte age at cryopreservation, laboratory expertise, and the vitrification and warming protocol, and some registry and sibling-oocyte data continue to suggest a modest advantage for fresh oocytes in selected settings (Rienzi *et al.*, 2010; Cobo and Diaz, 2011; Iussig *et al.*, 2019; Cornet-Bartolomé *et al.*, 2020). Nevertheless, the effectiveness of fertility preservation depends strongly on age at cryopreservation, with younger women consistently obtaining greater oocyte yields and higher cumulative live birth probabilities than their older counterparts (Doyle *et al.*, 2016; Cobo *et al.*, 2018, 2025; Kasaven *et al.*, 2022; Tsafrir *et al.*, 2022; Kirshenbaum *et al.*, 2025). Despite this evidence, patient awareness of the optimal timing for fertility preservation remains limited, and standardized counselling is often based on uniform oocyte number targets that may not reflect individual biological variability.

Over the past decade, artificial intelligence (AI) has been increasingly applied to medical imaging across reproductive medicine, particularly to improve embryo selection (Khosravi *et al.*, 2019). Although advancing maternal age is consistently associated with reduced oocyte competence (Galatidou *et al.*, 2024), oocyte quality varies substantially among patients of similar age and even within individual oocyte cohorts. Automated analysis of denuded metaphase II oocyte images using AI may therefore provide an objective, quantitative adjunct to age-based counselling for fertility preservation.

Individual oocyte competence varies substantially between women of similar age, yet fertility preservation counselling still relies largely on uniform, age-based oocyte-number targets. What has been lacking is a way to translate both maternal age and individual, objectively measured oocyte quality into a personalized, quantitative estimate of how many mature oocytes a given woman is likely to require. The present study therefore aimed to develop a biologically informed probabilistic model that integrates AI-derived oocyte morphology with age-dependent probabilities of blastulation and euploidy to estimate the minimum number of mature oocytes required to obtain three euploid blastocysts, and to apply this model to a large real-world fertility preservation cohort. Our aim in comparing model-derived requirements with observed yields is not to suggest that current retrieval practice is inadequate, since clinicians appropriately aim to retrieve as many oocytes as is safely feasible per cycle and to add storage cycles where indicated, but rather to provide an individualized benchmark that can support more personalized counselling, expectation-setting, and care planning.

## Materials and methods

### Study design and ethics

This retrospective cohort study was conducted at a private university-affiliated IVF centre between January 2012 and December 2024. The study was approved by the Research Ethics Committee of the Faculdade de Medicina de Jundiaí (opinion no. 7,488,073; CAAE 79784224.0.0000.5412). All participants provided written informed consent for the use of anonymized clinical data.

Three datasets were analysed. An autologous ICSI dataset served as the primary source for model development, providing oocyte-level information on blastocyst formation and MAGENTA™ score, and embryo-level information on age-specific euploidy rates. An ovum donation dataset was used to externally validate the MAGENTA effect on blastulation in a biologically distinct population of young donors without infertility-related indications. A fertility preservation cohort was used to compare model-derived biological requirements against real-world oocyte yields. The characteristics of the three datasets, including numbers of patients, cycles, oocytes and embryos, age distribution, and availability of PGT-A, are summarized in Supplementary Table S1, and the overall study design is shown schematically in Supplementary Fig. S1.

### Autologous ICSI dataset

This dataset comprised autologous ICSI cycles performed between January 2021 and December 2024. All oocytes were vitrified and subsequently warmed prior to ICSI. For each oocyte, maternal age, a 2D image captured prior to vitrification, and the corresponding blastocyst development outcome (blastocyst vs non-blastocyst) were recorded. A subset of embryos proceeded to trophectoderm biopsy and PGT-A. Two analytical subsets were extracted. The blastulation subset included 13 782 mature oocytes with complete information on MAGENTA score, maternal age, and blastocyst development outcome (1625 cycles from 1020 patients; maternal age 21–50 years). The euploidy subset included 3325 biopsied blastocysts with a conclusive PGT-A result (857 patients; maternal age 23–47 years). All images were analysed using the MAGENTA AI scoring system (Future Fertility, Toronto, Canada), which assigns a continuous 0–10 oocyte quality score. PGT-A was performed only in this autologous ICSI dataset; no PGT-A data were available for the ovum donation or fertility preservation datasets. Consequently, the external validation concerns the association between MAGENTA score and blastulation, and not the complete endpoint of euploid blastocyst formation.

### Ovum donation dataset

The ovum donation dataset comprised 1948 mature oocytes obtained from 333 ovum donation cycles performed in 271 donors between January 2020 and December 2024. For each oocyte, donor age, pre-vitrification MAGENTA score, and blastocyst development outcome were recorded. Donor age ranged from 18 to 36 years, and donors had no infertility-related indications. The distribution of oocytes across donor age groups and MAGENTA tertiles is presented in Supplementary Table S2. This dataset was used exclusively to externally validate the association between MAGENTA score and blastulation, providing a biologically contrasting population against which the autologous model could be benchmarked.

### Fertility preservation dataset

A total of 1647 elective fertility preservation cycles from 1100 patients performed between January 2012 and December 2024 were included. Patients were stratified into five clinically relevant maternal age categories (<30, 30–33, 34–36, 37–40, and >40 years), comprising 66, 311, 315, 640, and 315 cycles, respectively. A subset of 132 patients (12.0%) subsequently returned to use their cryopreserved oocytes; for these, laboratory and clinical outcomes of warming and ICSI cycles were also available. Due to the low number of warming cycles, four age groups were used in this analysis rather than the five groups applied elsewhere in the study.

### Controlled ovarian stimulation and oocyte retrieval

Controlled ovarian stimulation was performed with recombinant follicle-stimulating hormone (r-FSH; Gonal-F, Merck, Geneva, Switzerland; or Rekovelle, Ferring Pharmaceuticals, Saint-Prex, Switzerland) combined with a gonadotrophin-releasing hormone antagonist (cetrorelix acetate; Cetrotide, Merck). Follicular growth was monitored by transvaginal ultrasound from Day 4 of gonadotrophin administration. When adequate follicular growth and serum oestradiol levels were achieved, leuprolide acetate (Lupron, TAP Pharmaceuticals, North Chicago, IL, USA) was administered to trigger final follicular maturation. Oocytes were collected 35 h later by transvaginal ultrasound-guided aspiration, and metaphase II oocytes were vitrified.

### Oocyte vitrification, warming, and ICSI

Vitrification and warming were performed using the Cryotop method (Kuwayama, 2007). Within 3 h of oocyte retrieval, oocytes were exposed to equilibration solution followed by a 30-s exposure to vitrification solution. Individual oocytes were loaded onto a polypropylene strip in <0.1 µl of vitrification solution and plunged vertically into liquid nitrogen. For warming, the polypropylene strip was immersed directly into thawing solution at 37 °C for 1 min; oocytes were then transferred into dilution solution for 3 min and washed twice for 5 min each. All solutions and consumables were obtained from Kitazato (Tokyo, Japan). Within 3 h of warming, oocytes underwent ICSI following routine procedures (Palermo *et al.*, 1992).

### Embryo culture, biopsy, and genetic testing

Fertilization was confirmed ∼16 h after ICSI by the presence of two pronuclei. Embryos were cultured in 50-µl droplets of Global medium (LifeGlobal, Guilford, CT, USA) under paraffin oil under reduced oxygen tension (6% CO_2_, 5% O_2_, balance N_2_) at 37 °C for 5 days. Blastocysts were graded from 1 to 6 based on expansion and hatching status, with full, expanded, hatching, and hatched blastocysts classified as complete blastocysts, a classification based on the degree of expansion and independent of the good inner cell mass and trophectoderm criteria used to select blastocysts for biopsy. On Day 3, assisted hatching was performed with laser pulsing (OCTAX Laser Shot; MTG Medical Technology, Altdorf, Germany). On Day 5, blastocysts with good-quality inner cell mass and trophectoderm were biopsied; trophectoderm cells were analysed by next-generation sequencing using the VeriSeq NGS platform (Illumina, San Diego, CA, USA), as previously described (Munné *et al.*, 2017). Embryos were classified as euploid, aneuploid, or mosaic based on copy number variation thresholds. The same embryo biopsy criteria, PGT-A platform (Veriseq NGS), and copy-number classification thresholds were applied consistently throughout the study period (2021–2024).

### Statistical analysis

A probabilistic framework was developed to estimate the minimum number of mature oocytes required to achieve at least three euploid blastocysts with a probability of 90% or greater, by maternal age and AI-derived oocyte quality. The framework combined two logistic regression models fitted to the autologous ICSI dataset, assuming conditional independence between blastocyst formation and euploidy. Blastulation and euploidy were modelled separately, rather than as a single euploid-blastocyst outcome, because the two outcomes were observed at different levels and in different subsets: blastocyst development was recorded for all mature oocytes (n = 13 782), whereas euploidy could be determined only for biopsied blastocysts (n = 3325), since non-blastocysts and blastocysts not selected for biopsy have no PGT-A result. This two-stage decomposition allowed each component to be estimated from the largest relevant sample, with the MAGENTA (oocyte-quality) effect on blastulation and the age effect on euploidy each estimated from the most informative data. The assumption of conditional independence between blastocyst formation and euploidy, given maternal age and oocyte quality, is biologically reasonable because embryonic aneuploidy arises predominantly from age-related meiotic errors established at the oocyte level, largely independently of subsequent blastocyst development; any residual dependence would tend to modestly overestimate cumulative success and is acknowledged as a limitation.

#### Blastulation model

Blastocyst formation (blastocyst vs non-blastocyst), defined as development to a blastocyst of any morphological grade and not restricted to blastocysts meeting criteria for biopsy, was modelled as a function of MAGENTA score and maternal age, both as continuous predictors. Cluster-robust standard errors were applied at the cycle level using a sandwich variance estimator with a small-sample correction to account for multiple oocytes from the same stimulation cycle.

#### Euploidy model

Euploidy (euploid vs aneuploid, with mosaic embryos excluded from the primary analysis and examined in sensitivity analyses) was modelled as a function of maternal age, entered as a cubic polynomial centred at 35 years to accommodate the non-linear age-related decline. Cluster-robust standard errors were applied at the patient level to account for multiple embryos from the same individual.

#### External validation

The blastulation model was validated in the ovum donation dataset in two ways. First, the model developed in the autologous ICSI cohort was applied directly to the ovum donation dataset without any re-estimation of its coefficients, and out-of-sample discrimination was quantified by the area under the receiver operating characteristic curve. Second, the model was refitted within the ovum donation dataset to assess whether the direction and magnitude of the MAGENTA coefficient were reproduced.

#### MAGENTA tertile categorization

For clinical presentation, MAGENTA scores were classified into three categories (Low, Intermediate, High) using tertile cut-offs (3.70 and 8.40) derived from the ovum donation dataset distribution. The median MAGENTA within each tertile (1.10, 6.40, and 9.50) was used as the representative value for prediction. These cut-offs were applied uniformly to the autologous ICSI and ovum donation datasets, in which MAGENTA scores were available; MAGENTA scores were not available for the fertility preservation dataset.

#### Oocytes required

For each maternal age × MAGENTA stratum, the per-oocyte probability of yielding a euploid blastocyst was computed as the product of the predicted blastulation probability and the predicted age-specific euploidy probability. The minimum integer number of mature oocytes required to reach a probability of 90% or greater of obtaining at least three euploid blastocysts was determined using a binomial probability model. Ninety-five per cent CIs around the required oocyte number were generated by cluster-resampled non-parametric bootstrapping (500 replicates), in which cycles (blastulation) and patients (euploidy) were resampled with replacement. Representative maternal ages for each age category were 28, 32, 35, 38.5, and 42 years for <30, 30–33, 34–36, 37–40, and >40 years, respectively.

The proportion of patients expected to reach the model-derived targets was approximated using a negative-binomial distribution for the cumulative number of mature oocytes per patient. The distribution was parameterized from the observed cumulative mean in each maternal age group and a coefficient of variation of ∼1, consistent with the dispersion observed in the fertility preservation cohort, and the expected proportion was taken as the fraction of that distribution equalling or exceeding the corresponding model-derived requirement. Because this approximation was derived from aggregate cohort statistics rather than from patient-level records, the resulting proportions are indicative rather than exact.

All analyses were performed in Python 3.12 (Python Software Foundation, Beaverton, OR, USA) using the scikit-learn (Pedregosa *et al.*, 2011) and scipy (Virtanen *et al.*, 2020) libraries. A two-sided *P* < 0.05 was considered statistically significant.

## Results

### Patient and cycle characteristics

Between 2012 and 2024, 1647 elective fertility preservation cycles were performed in 1100 patients. Of these, 132 patients (12.0%) subsequently returned to use their cryopreserved oocytes. Patients were older at the time of oocyte warming than at cryopreservation. Demographic and clinical characteristics of fertility preservation cycles are presented in Table 1; the corresponding characteristics for the subset of warming cycles are provided in Supplementary Table S3. Outcomes of warming and ICSI cycles across maternal age groups are summarized in Supplementary Table S4. Due to the low number of warming cycles (n = 132), four age groups were used in this analysis rather than the five groups applied elsewhere in the study. Oocyte survival and clinical pregnancy rates were numerically lower in women older than 40 years than in the younger groups, whereas fertilization, blastulation, and implantation rates were similar across age groups; these outcomes are reported descriptively. Miscarriage analysis was not performed due to the low event count (n = 5), which occurred predominantly in older age groups.

**Table 1.** Demographic and clinical characteristics of patients undergoing elective fertility preservation cycles (n = 1647).

Variable	Mean	SD
Maternal age, years	37.0	3.8
Body mass index, kg/m²	23.9	3.7
Aspirated follicles, n	12.9	11.1
Retrieved oocytes, n	9.2	8.7
Oocyte retrieval rate, %	67.3	25.5
Mature (MII) oocytes retrieved, n	6.6	6.6
Oocyte maturation rate, %	69.9	25.8
Storage duration, months	19.5	22.5

Values are expressed as mean and SD. Oocyte retrieval rate is the number of retrieved oocytes as a proportion of aspirated follicles; oocyte maturation rate is the number of mature oocytes as a proportion of retrieved oocytes. No statistical comparisons were performed in this table. MII, metaphase II.

### Blastulation model

Among the 13 782 mature oocytes from autologous ICSI cycles, 6939 (50.3%) developed into blastocysts. MAGENTA score and maternal age were independently associated with blastulation. Each additional MAGENTA point was associated with a 19% increase in the odds of blastocyst formation (OR 1.19, 95% CI 1.18–1.21; *P* < 0.001), whereas each additional year of maternal age was associated with a 6% decrease in the odds of blastulation (OR 0.94, 95% CI 0.93–0.96; *P* < 0.001). The MAGENTA effect on blastulation was replicated in the ovum donation dataset, with preserved direction and statistical significance (OR 1.11 per point, 95% CI 1.08–1.14), supporting the reproducibility of the oocyte quality component of the model in a biologically distinct population. When the autologous model was applied to the ovum donation dataset without refitting, it retained discrimination (out-of-sample AUC 0.60); absolute blastulation was lower in donation cycles (39.3% vs 50.3%), consistent with the different sperm source, so that the MAGENTA association, rather than the absolute rate, transported across populations.

### Euploidy model

Among the 3325 biopsied embryos with a conclusive PGT-A result, 1207 (36.3%) were euploid. Euploidy declined non-linearly with maternal age, from 68.6% in women younger than 30 years to 54.1% at 30–33 years, 50.3% at 34–36 years, 34.0% at 37–40 years, and 19.3% at older than 40 years. Logistic regression with a cubic age term confirmed a highly significant age-related decline (*P* < 0.001 for linear and quadratic terms). Mosaic embryos (n = 201) were excluded from the primary analysis. Classifying them as non-euploid or as euploid yielded overall euploidy rates of 34.2% and 39.9%, respectively.

### Model performance

Model performance was assessed by discrimination, calibration, and observed-versus-predicted comparison (Supplementary Fig. S2). For the blastulation model, discrimination was modest (area under the receiver operating characteristic curve [AUC] 0.66) and calibration was excellent, with predicted probabilities closely tracking observed blastulation across deciles of predicted risk and calibration-in-the-large in exact agreement (predicted 50.3% vs observed 50.3%; Brier score 0.23). For the euploidy model, discrimination was similarly modest (AUC 0.67) with excellent calibration, predicted euploidy closely matching observed euploidy across deciles of predicted risk and across maternal-age groups (Table 2), and calibration-in-the-large in exact agreement (predicted 36.3% vs observed 36.3%; Brier score 0.21). Modest discrimination is expected at the level of an individual oocyte or embryo, where blastulation and euploidy are strongly stochastic; for a probabilistic tool that aggregates per-oocyte probabilities across a cohort of oocytes, calibration is the relevant property, and calibration was excellent for both models.

**Table 2.** Observed and model-predicted euploidy by maternal age group.

Maternal age group	Observed euploidy, %	Model-predicted euploidy, %
<30 years	68.6^a^	58.7
30–33 years	54.1	58.3
34–36 years	50.3	49.9
37–40 years	34.0	34.1
>40 years	19.3	18.1

Observed euploidy is the proportion of biopsied blastocysts with a euploid result on preimplantation genetic testing for aneuploidy; model-predicted euploidy is the probability estimated by the age-based logistic model. No statistical comparisons were performed in this table. ^a^The <30-year group comprised a small number of biopsied embryos (n = 51) and is therefore subject to substantial sampling variability.

### Model-estimated oocyte requirements

Integration of the blastulation and euploidy models yielded estimates of the minimum number of mature oocytes required to achieve at least three euploid blastocysts at a probability of 90% or greater, stratified by maternal age and AI-derived oocyte quality (Fig. 1; Table 3). The estimated requirement increased with advancing age and decreasing oocyte quality. For women younger than 30 years, the estimated requirement was 18 (95% CI 15–22), 11 (10–13), and 9 (8–11) mature oocytes for Low, Intermediate, and High MAGENTA, respectively. Corresponding estimates were 24 (21–27), 14 (13–16), and 11 (10–13) for 30–33 years; 32 (29–34), 18 (17–19), and 14 (13–15) for 34–36 years; 52 (47–57), 29 (27–31), and 22 (21–24) for 37–40 years; and 121 (104–146), 65 (57–77), and 49 (43–58) for older than 40 years. The increase was particularly pronounced in the >40-year Low-MAGENTA stratum, reflecting the combined effects of reduced blastulation and markedly decreased euploidy. Sensitivity analyses varying the reproductive target (2, 3, or 4 euploid blastocysts) and confidence threshold (80%, 90%, or 95%) confirmed that the direction and relative magnitude of the age and MAGENTA effects were preserved across all scenarios (Fig. 2; Supplementary Table S5).

**Figure 1.**
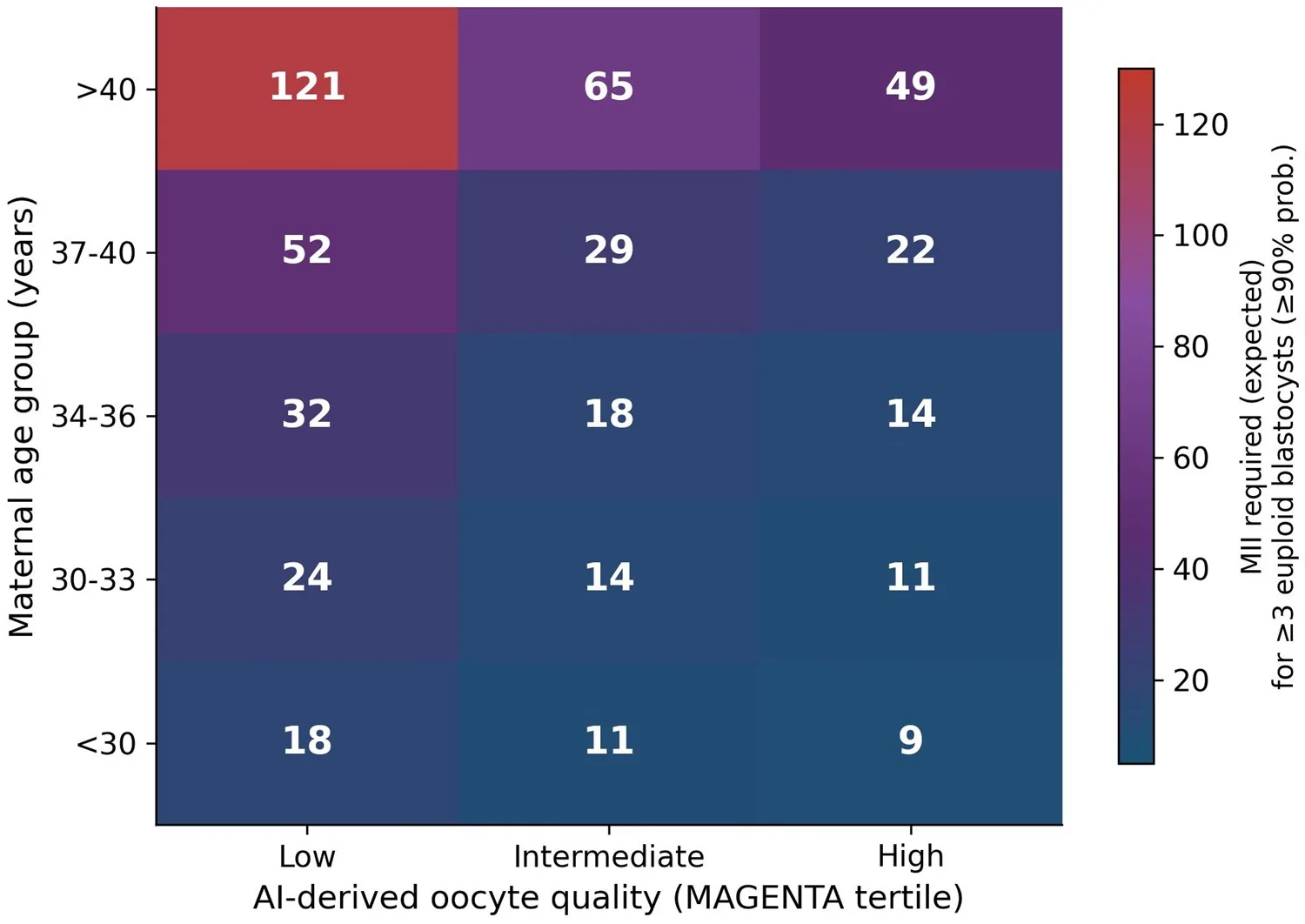
**Model-estimated number of mature oocytes required to achieve at least three euploid blastocysts.** Heat-map showing the minimum integer number of mature oocytes required to obtain at least three euploid blastocysts with a probability of 90% or greater, stratified by maternal age group (rows) and AI-derived oocyte quality (MAGENTA tertile; columns). Colour intensity reflects the magnitude of the estimated requirement, from lower (teal) to higher (red) values. AI, artificial intelligence; MAGENTA, an artificial-intelligence-derived oocyte quality score (0–10 scale) generated from a static image of the mature oocyte; MII, metaphase II.

**Figure 2.**
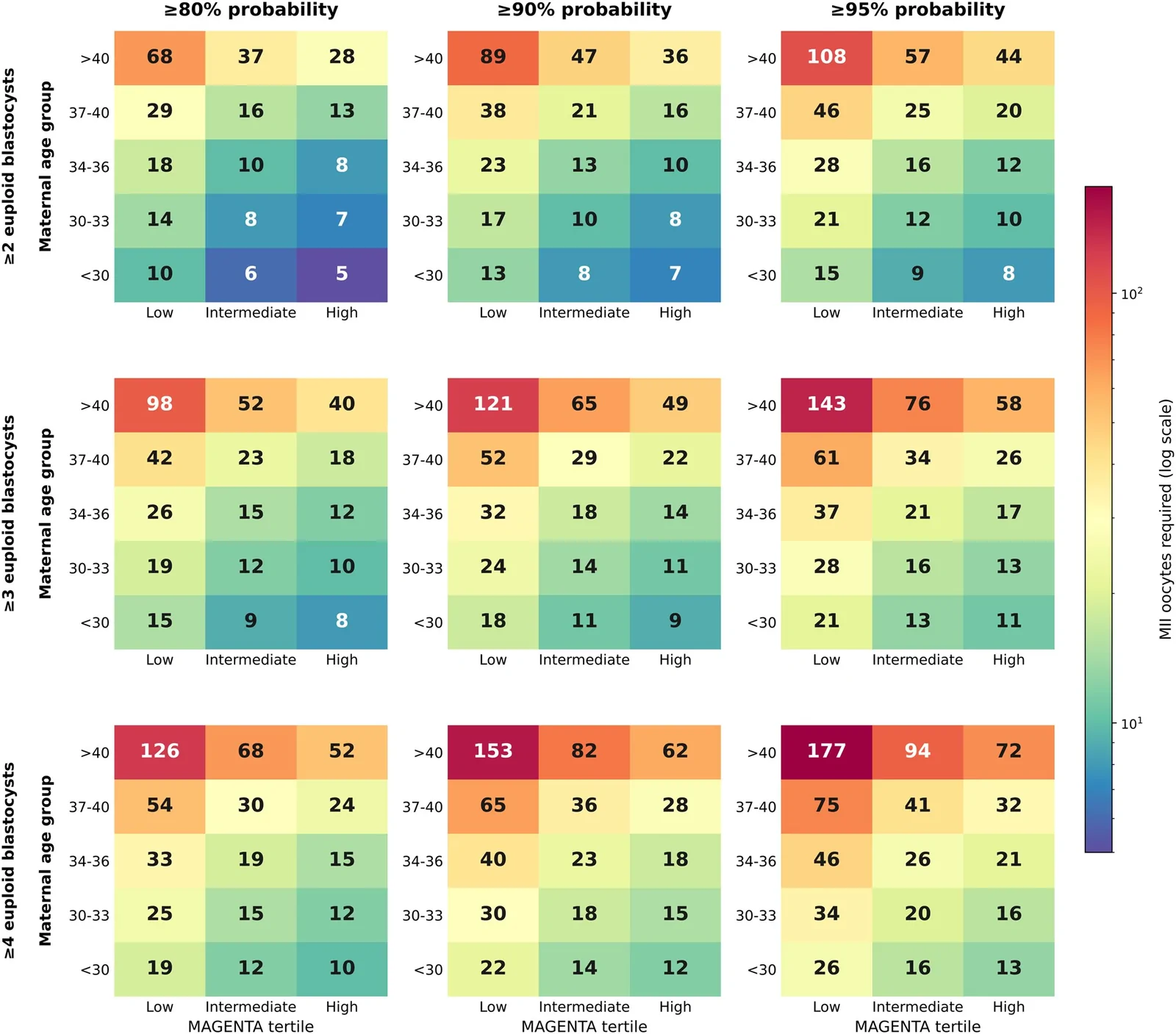
**Sensitivity analysis of the estimated number of mature oocytes required, across alternative reproductive targets and confidence thresholds.** Nine heat-maps showing the estimated requirement across reproductive targets of two, three, or four euploid blastocysts (rows) and confidence thresholds of 80%, 90%, or 95% (columns), stratified by maternal age and MAGENTA tertile. Across all nine scenarios, the direction and relative magnitude of the age and MAGENTA effects are preserved, supporting the robustness of the primary analysis (≥3 euploid blastocysts at ≥90% probability; central panel). MAGENTA, an artificial-intelligence-derived oocyte quality score (0–10 scale) generated from a static image of the mature oocyte; MII, metaphase II.

**Table 3.** Model-estimated probability of blastulation, euploidy, and their combined probability per mature oocyte, and the minimum number of mature oocytes required to achieve at least three euploid blastocysts at a probability of 90% or greater, by maternal age and MAGENTA tertile.

Maternal age	MAGENTA tertile	*P*(blast)	*P*(eupl)	*P*(combined)	N (95% CI)
<30	Low	0.391	0.684	0.267	18 (15–22)
	Intermediate	0.616	0.684	0.421	11 (10–13)
	High	0.741	0.684	0.507	9 (8–11)
30–33	Low	0.341	0.541	0.184	24 (21–27)
	Intermediate	0.560	0.541	0.303	14 (13–16)
	High	0.692	0.541	0.374	11 (10–13)
34–36	Low	0.281	0.503	0.141	32 (29–34)
	Intermediate	0.490	0.503	0.247	18 (17–19)
	High	0.626	0.503	0.315	14 (13–15)
37–40	Low	0.207	0.340	0.070	52 (47–57)
	Intermediate	0.387	0.340	0.132	29 (27–31)
	High	0.520	0.340	0.177	22 (21–24)
>40	Low	0.150	0.193	0.029	121 (104–146)
	Intermediate	0.298	0.193	0.058	65 (57–77)
	High	0.420	0.193	0.081	49 (43–58)

MAGENTA is an artificial-intelligence-derived oocyte quality score (0–10 scale) generated from a static image of the mature oocyte; tertile cut-offs were 3.70 and 8.40. *P*(blast) is the per-oocyte probability of blastocyst formation estimated by the logistic model including MAGENTA score and maternal age; *P*(eupl) is the age-specific probability of euploidy in biopsied blastocysts; *P*(combined) = *P*(blast) × *P*(eupl). N is the minimum number of mature oocytes required to achieve at least three euploid blastocysts with a probability of 90% or greater. CIs were obtained by cluster-resampled bootstrapping (500 replicates). No statistical comparisons were performed in this table.

### Comparison with real-world fertility preservation practice

The median number of mature oocytes cryopreserved per fertility preservation cycle declined progressively with maternal age: 8.3, 8.1, 7.3, 6.1, and 4.0 for women aged <30, 30–33, 34–36, 37–40, and >40 years, respectively. When contrasted with model-derived biological requirements, a progressive divergence between observed yield and estimated need was observed and widened with age (Fig. 3). Among women younger than 30 years, the median observed yield (8.3 oocytes) approached the model-estimated requirement for High MAGENTA (9 oocytes) but remained below requirements for Intermediate (11) and Low (18) MAGENTA. From 34 to 36 years onward, the median observed yield fell below the estimated requirement across all MAGENTA categories, with the discrepancy widening progressively. Among women older than 40 years, the median of four oocytes per cycle was 12- to 30-fold lower than the model-estimated requirement across MAGENTA categories.

**Figure 3.**
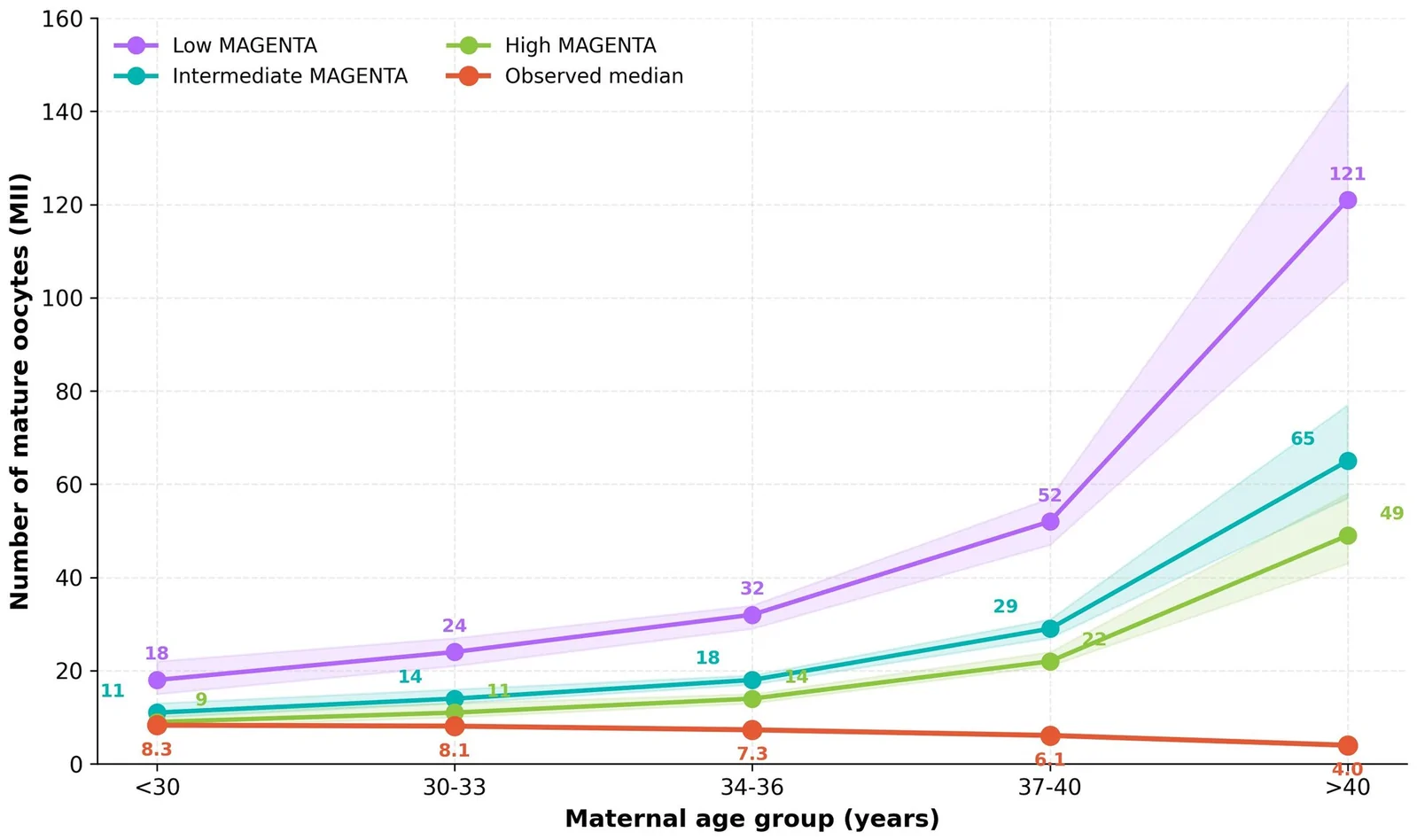
**Comparison of observed and model-estimated number of mature oocytes per fertility preservation cycle, by maternal age.** Line plot showing the observed median number of mature oocytes cryopreserved per cycle (orange) and the model-estimated minimum number of mature oocytes required to achieve at least three euploid blastocysts at a probability of 90% or greater, stratified by AI-derived oocyte quality (Low MAGENTA in purple, Intermediate in teal, High in green). Shaded bands represent 95% bootstrap CIs around the estimated requirements. The discrepancy between observed yield and estimated requirement widens progressively with age. CIs were obtained by cluster-resampled bootstrapping (500 replicates). MAGENTA, an artificial-intelligence-derived oocyte quality score (0–10 scale) generated from a static image of the mature oocyte; MII, metaphase II.

The cumulative yield per patient was 12.4, 12.1, 10.9, 9.1, and 6.0 mature oocytes for women aged <30, 30 to 33, 34 to 36, 37 to 40, and >40 years, respectively. Even on a cumulative per-patient basis, the estimated yield met the High-MAGENTA target only in women younger than 34 years and fell below all three MAGENTA-based targets from 34 to 36 years onward. Under a negative-binomial approximation consistent with the observed dispersion (coefficient of variation of ∼1), the estimated proportion of patients reaching the target of at least three euploid blastocysts declined steeply with age; for the High-MAGENTA target, it was ∼51%, 43%, 30%, 10%, and 0% for women aged <30, 30 to 33, 34 to 36, 37 to 40, and >40 years, respectively. These figures are approximations derived from aggregate cohort statistics rather than from patient-level records, and should be interpreted with caution.

## Discussion

Elective fertility preservation has become an established strategy in contemporary reproductive medicine, yet important uncertainties remain regarding the optimal age at oocyte vitrification and the number of oocytes required to achieve a reasonable likelihood of live birth. In the present study, we developed a probabilistic model to estimate the number of mature oocytes required to obtain three euploid blastocysts, a threshold associated with a high cumulative probability of pregnancy (Ata *et al.*, 2021; Pirtea *et al.*, 2021), based on maternal age and AI-derived oocyte quality, and applied this model to a large real-world fertility preservation cohort to evaluate whether current clinical practice achieves biologically adequate oocyte yields. Our findings suggest that real-world fertility preservation strategies frequently fall short of biologically informed targets, particularly when vitrification is undertaken after the mid-thirties.

Each additional MAGENTA point was associated with a 19% increase in the odds of blastocyst formation, and the age-related decline in euploidy further raised the required oocyte number. When integrated, these biological components produced marked age- and quality-dependent estimates: a woman younger than 30 years with High MAGENTA was estimated to need nine oocytes, whereas a woman older than 40 years with Low MAGENTA was estimated to need 121 to achieve the same reproductive target. The estimated requirement was broadly similar between High and Intermediate MAGENTA categories but substantially greater for Low MAGENTA, and this divergence widened with age. These observations suggest that AI-derived oocyte quality contributes meaningfully to reproductive potential, particularly at the extremes of reproductive ageing.

The MAGENTA effect on blastulation was replicated in the ovum donation dataset, an independent population of young donors without infertility-related indications, supporting the robustness of the oocyte quality component and suggesting that MAGENTA captures an intrinsic biological property of the oocyte rather than a feature specific to any one clinical context.

Application of the model to the fertility preservation cohort revealed a progressive discrepancy between biological requirements and cryopreserved oocyte numbers. Women younger than 30 years vitrified numbers approaching model estimates for High MAGENTA but below those for Intermediate and Low MAGENTA. From 34 to 36 years onward, the median cryopreserved number was below the estimated requirement across all MAGENTA categories. Among women older than 40 years, the median of four oocytes per cycle was 12- to 30-fold lower than model-estimated requirements, indicating that fertility preservation at advanced reproductive ages rarely achieves a biologically adequate yield within a single cycle.

Our findings align with prior evidence that earlier fertility preservation is associated with higher cumulative live birth probability (Kasaven *et al.*, 2022; Tsafrir *et al.*, 2022; Cobo *et al.*, 2025; Kirshenbaum *et al.*, 2025). The present results extend this evidence by providing a biological framework that links increasing oocyte requirements to age-related reductions in euploidy, while showing that AI-derived oocyte quality adds prognostic refinement within age groups.

In the warming-cycle analysis, lower oocyte survival and pregnancy rates were observed among older women. Although high post-warming survival rates are widely reported, age-related deterioration in oocyte competence may contribute to reduced reproductive efficiency. Cryopreservation may induce structural and functional alterations, with susceptibility to cryoinjury influenced by membrane permeability, osmotic tolerance, and sensitivity to cryoprotectant exposure (Moussa *et al.*, 2014).

This study has important strengths. The probabilistic model was developed using a large autologous ICSI cohort spanning a wide age range (21–50 years), with 13 782 oocytes in the blastulation model and 3325 biopsied embryos in the euploidy model. The MAGENTA effect on blastulation was externally validated in a biologically independent donor cohort. Cluster-robust standard errors and cluster-resampled bootstrapping enhance the statistical rigour of the reported estimates. Application of the model to a real-world fertility preservation cohort spanning more than a decade allowed assessment of clinical practice beyond theoretical projections.

The predictive probabilities reported here are conditioned on the clinical and laboratory context in which the datasets were generated, including patient selection and infertility indications, ovarian stimulation, vitrification and warming, ICSI practice, embryo culture conditions, blastocyst grading and biopsy criteria, the PGT-A platform, and the population used to develop and validate the AI system. Differences in any of these factors between laboratories may shift absolute blastulation and euploidy rates and, consequently, the absolute oocyte numbers estimated by the model. Consistent with this, our external validation showed that the MAGENTA effect on blastulation reproduced in a biologically distinct donor population, whereas absolute blastulation rates differed. The absolute estimates should therefore be regarded as specific to comparable settings, while the direction and relative magnitude of the age and MAGENTA effects are expected to be more generalizable. Larger multicentre datasets will be required to determine whether these estimates are directly transferable across laboratories, countries, and patient populations, and to recalibrate the model where necessary. Multicentre validation and recalibration therefore represent an essential next step towards broad clinical implementation of the present approach.

MAGENTA and similar AI-derived oocyte-quality tools are best regarded as adjuncts to counselling rather than as deterministic predictors of reproductive success. The model estimates the number of oocytes required to obtain euploid blastocysts and does not directly predict live birth, which depends further on implantation, endometrial and obstetric factors, and the sperm contribution. These estimates should therefore inform, rather than replace, individualized clinical judgement, and counselling should avoid both false reassurance and undue pessimism.

The observation that women older than 35 years, and particularly those older than 40, frequently cryopreserve far fewer oocytes than the model-estimated requirement has direct implications for counselling. It indicates that a single stimulation cycle at advanced reproductive age is unlikely to provide a biologically adequate oocyte yield, and that additional cycles may be required, with corresponding implications for cost, time, and emotional burden. Rather than defining a universal minimum number of oocytes, these estimates support earlier and more individualized counselling, so that women can make informed decisions about the timing of fertility preservation and hold realistic expectations of the number of cycles likely to be needed.

Several limitations should be acknowledged. Blastulation and euploidy probabilities were derived from autologous ICSI cycles in patients with infertility-related indications that may differ from those electing fertility preservation. Extrapolation to elective cryopreservation candidates therefore assumes that per-oocyte biological competence, conditional on age and AI-derived quality, is transportable across these populations; although the MAGENTA effect was validated in donors, residual biological differences cannot be excluded. The probabilistic framework assumes conditional independence between blastocyst formation and euploidy, which may modestly overestimate cumulative success in some contexts. Finally, the model estimates the number of oocytes required to produce euploid blastocysts but does not directly model implantation, clinical pregnancy, or live birth, which are further conditional on endometrial and obstetric factors. In addition, the absence of MAGENTA scoring in the fertility preservation cohort precludes stratifying the proportion of patients reaching the target by patients’ own oocyte quality. Blastocyst formation was defined as development to a blastocyst of any grade, whereas euploidy was assessed only in blastocysts selected for biopsy on the basis of good-quality inner cell mass and trophectoderm; the euploidy rate therefore applies to biopsy-quality blastocysts and may differ from that of all blastocysts. Three euploid blastocysts is therefore a clinically meaningful but intermediate benchmark, and the estimates should be interpreted as oocyte requirements for obtaining euploid blastocysts rather than as predictions of live birth, which depends additionally on embryo-endometrial interaction, the sperm contribution, and patient-specific characteristics. Mosaic embryos, which may retain reproductive potential in selected circumstances, were excluded from the primary analysis given their debated clinical interpretation; sensitivity analyses classifying them as euploid or as non-euploid changed the overall euploidy rate only modestly and did not materially alter the estimates.

In conclusion, both maternal age and AI-derived oocyte quality strongly influence the number of mature oocytes required to achieve an adequate yield of euploid blastocysts. Integration of AI-based oocyte assessment with age-specific euploidy suggests that many women undergoing fertility preservation accumulate fewer oocytes than biologically required to achieve a high likelihood of future reproductive success. These estimates are intended as a counselling and care-planning aid rather than as a critique of clinical practice. Framed this way, the divergence between observed yields and model-derived requirements provides an individualized basis for discussing how many oocytes, and where relevant how many stimulation cycles, a given woman is likely to need, and reinforces the value of earlier, more personalized counselling. Incorporating AI-derived oocyte quality alongside age-related chromosomal competence may enable more individualized counselling and help mitigate overly optimistic expectations, particularly in women pursuing oocyte cryopreservation at more advanced reproductive ages.

## Supplementary Material

hoag090_Supplementary_Data

## Data Availability

The data underlying this article cannot be shared publicly owing to privacy and confidentiality of patient information. Aggregate data and the analytical code are available from the corresponding author upon reasonable request, subject to institutional data-sharing policies and ethical approval.
