# Supplementary material for: Are women cryopreserving enough oocytes for fertility preservation? A probabilistic model integrating AI-based oocyte morphology and age-dependent euploidy
Source: Hum Reprod Open. 2026 Oct 14;2026(4):hoag090. doi: 10.1093/hropen/hoag090 (PMC13690086; doi:10.1093/hropen/hoag090)
Supplement: Supplementary file 1 [file hoag090_supplementary_data.zip › Braga__HROpen__R2__Supplementary_Figure_S1.pdf]

Supplementary Figure S1. Study design and contribution of the three datasets

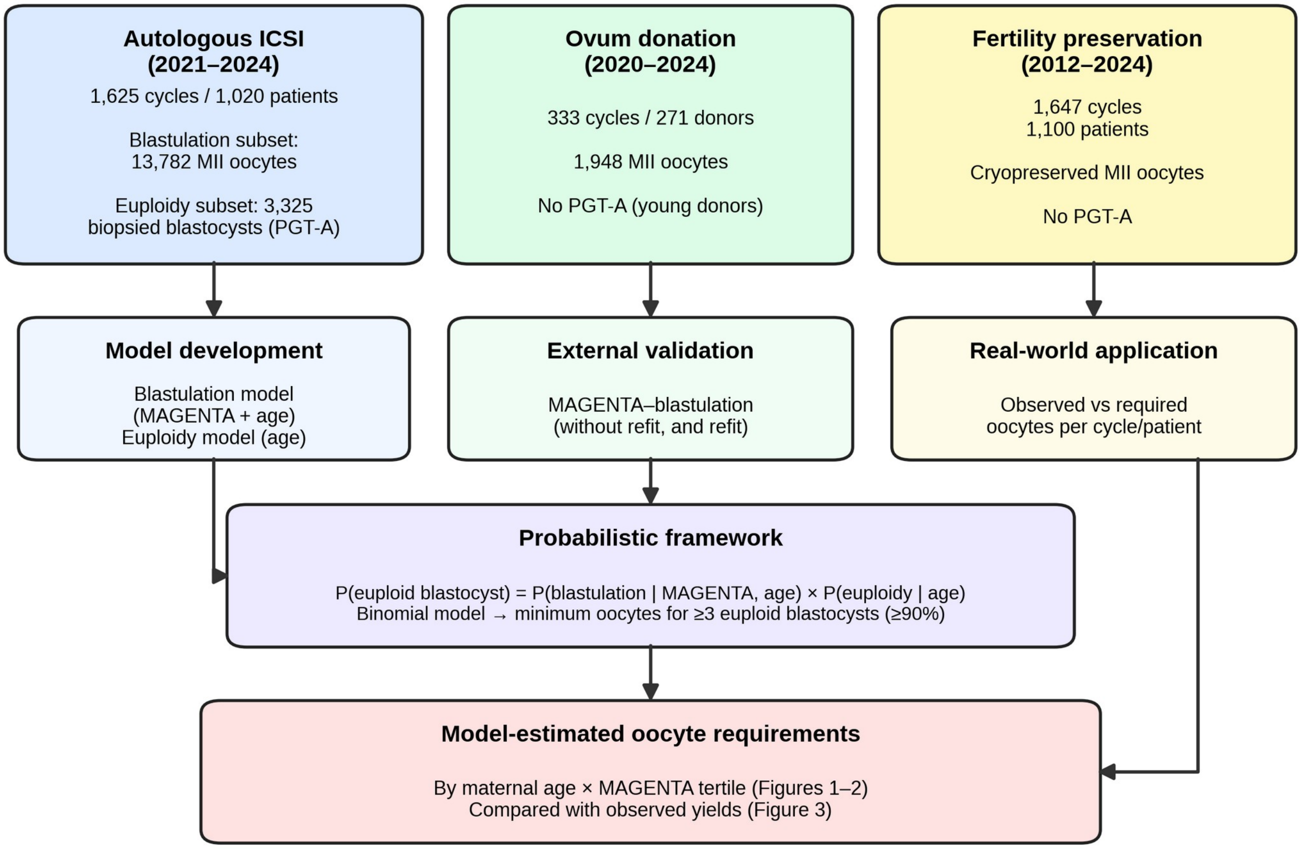

Supplementary Figure S1. Study design and contribution of the three datasets. Schematic overview of how each dataset contributed to the study: the autologous ICSI dataset (development of the blastulation and euploidy models), the ovum donation dataset (external validation of the association between MAGENTA score and blastulation), and the fertility preservation dataset (real-world application), each feeding into the probabilistic framework and the model-estimated oocyte requirements. No statistical tests are presented in this figure. ICSI, intracytoplasmic sperm injection; MAGENTA, an artificial-intelligence-derived oocyte quality score (0–10 scale) generated from a static image of the mature oocyte; MII, metaphase II; PGT-A, preimplantation genetic testing for aneuploidy.
