# Supplementary material for: Are women cryopreserving enough oocytes for fertility preservation? A probabilistic model integrating AI-based oocyte morphology and age-dependent euploidy
Source: Hum Reprod Open. 2026 Oct 14;2026(4):hoag090. doi: 10.1093/hropen/hoag090 (PMC13690086; doi:10.1093/hropen/hoag090)
Supplement: Supplementary file 1 [file hoag090_supplementary_data.zip › Braga__HROpen__R2__Supplementary_Figure_S2.pdf]

Supplementary Figure S2. Calibration of the blastulation and euploidy models

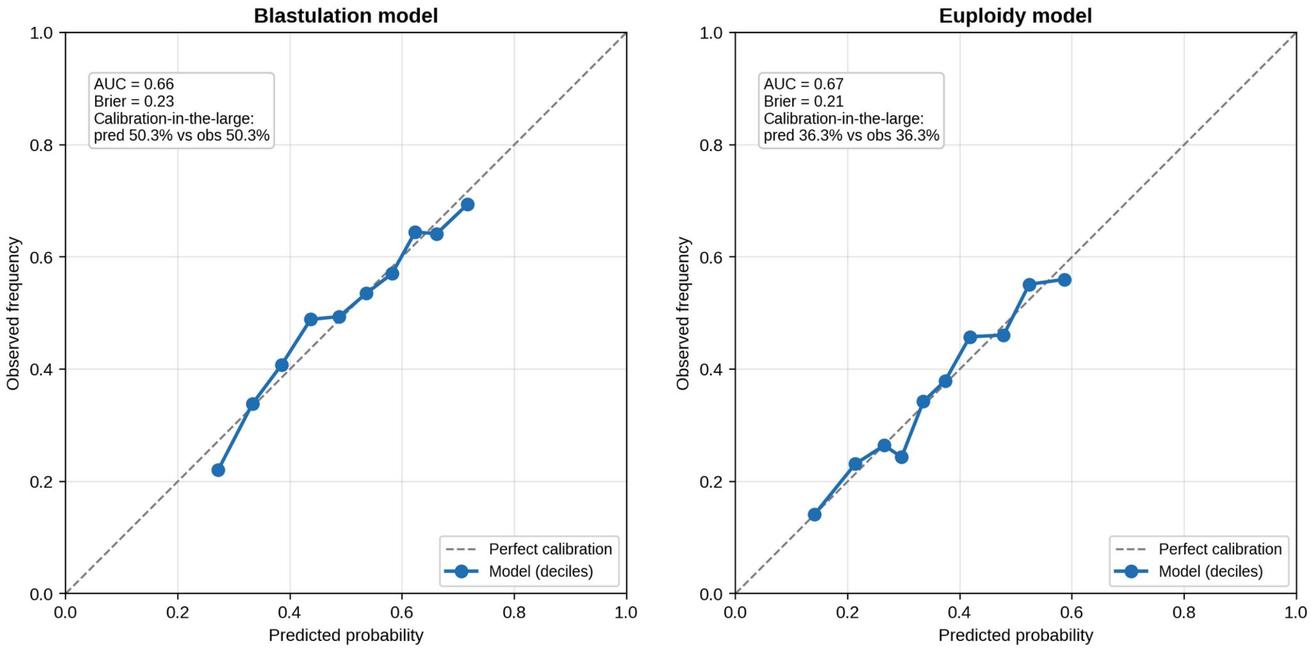

Supplementary Figure S2. Calibration of the blastulation and euploidy models. Calibration plots showing predicted versus observed probabilities across deciles of predicted risk for the blastulation model (left) and the euploidy model (right); the dashed line indicates perfect calibration. Insets report the area under the receiver operating characteristic curve, the Brier score, and calibration-in-the-large (mean predicted versus mean observed probability). AUC, area under the receiver operating characteristic curve.
