# Supplementary material for: Are women cryopreserving enough oocytes for fertility preservation? A probabilistic model integrating AI-based oocyte morphology and age-dependent euploidy
Source: Hum Reprod Open. 2026 Oct 14;2026(4):hoag090. doi: 10.1093/hropen/hoag090 (PMC13690086; doi:10.1093/hropen/hoag090)
Supplement: Supplementary file 1 [file hoag090_supplementary_data.zip › Braga__HROpen__R2__Supplementary_Table_S2.docx]

**Supplementary Table S2.** Distribution of mature oocytes by donor age and MAGENTA tertile in the ovum donation dataset.

| **Donor age, years** | **Low (≤3.70)** | **Intermediate (3.71–8.40)** | **High (>8.40)** | **Total** |
| --- | --- | --- | --- | --- |
| <30 | 444 | 472 | 475 | 1,391 |
| 30–33 | 196 | 161 | 153 | 510 |
| 34–36 | 20 | 14 | 13 | 47 |
| Total | 660 | 647 | 641 | 1,948 |

Bold text is used for column headings only. Values are the number of mature oocytes in each donor age × MAGENTA tertile stratum. No statistical comparisons were performed in this table. MAGENTA scores are reported on a 0–10 scale; tertile cut-offs (3.70 and 8.40) were derived from the empirical distribution in this dataset and applied also to the autologous ICSI dataset. The ovum donation dataset comprised 1,948 oocytes from 333 cycles performed in 271 donors aged 18–36 years. MAGENTA, an artificial-intelligence-derived oocyte quality score generated from a static image of the mature oocyte.
