# Supplementary material for: Are women cryopreserving enough oocytes for fertility preservation? A probabilistic model integrating AI-based oocyte morphology and age-dependent euploidy
Source: Hum Reprod Open. 2026 Oct 14;2026(4):hoag090. doi: 10.1093/hropen/hoag090 (PMC13690086; doi:10.1093/hropen/hoag090)
Supplement: Supplementary file 1 [file hoag090_supplementary_data.zip › Braga__HROpen__R2__Supplementary_Table_S4.docx]

**Supplementary Table S4.** Survival rates, laboratory results, and clinical outcomes of warming / ICSI cycles by maternal age.

| **Variable** | **<30** | **30–35** | **36–40** | **>40** |
| --- | --- | --- | --- | --- |
| Patients, n | 24 | 33 | 42 | 33 |
| Survival rate, % | 90.7 ± 33.6 | 81.4 ± 40.4 | 83.4 ± 35.5 | 72.3 ± 34.9 |
| Fertilisation rate, % | 78.9 ± 2.7 | 75.7 ± 3.2 | 73.2 ± 8.6 | 75.9 ± 2.8 |
| Blastulation rate, % | 47.9 ± 14.2 | 42.9 ± 4.8 | 41.3 ± 4.5 | 39.5 ± 5.6 |
| Implantation rate, % | 37.5 ± 14.5 | 40.8 ± 7.4 | 48.7 ± 6.4 | 23.4 ± 11.4 |
| Clinical pregnancy rate, % (n/N) | 50.0 (12/24) | 56.0 (14/25) | 46.3 (19/41) | 21.4 (6/28) |

Bold text is used for column headings only. Values are expressed as mean and standard deviation, except for clinical pregnancy rate, which is expressed as % (n/N). Maternal age is given in years. Because of the limited number of warming / ICSI cycles (n = 132), patients were grouped into four maternal age categories in this analysis rather than the five categories used elsewhere in the study. Data are reported descriptively. ICSI, intracytoplasmic sperm injection.
