# Supplementary material for: Are women cryopreserving enough oocytes for fertility preservation? A probabilistic model integrating AI-based oocyte morphology and age-dependent euploidy
Source: Hum Reprod Open. 2026 Oct 14;2026(4):hoag090. doi: 10.1093/hropen/hoag090 (PMC13690086; doi:10.1093/hropen/hoag090)
Supplement: Supplementary file 1 [file hoag090_supplementary_data.zip › Braga__HROpen__R2__Supplementary_Table_S5.docx]

**Supplementary Table S5.** Sensitivity analysis: minimum number of mature oocytes required to achieve alternative reproductive targets at alternative confidence thresholds, by maternal age and MAGENTA tertile.

| **Maternal age, years** | **MAGENTA tertile** | **≥2 euploid blastocysts** | | | **≥3 euploid blastocysts** | | | **≥4 euploid blastocysts** | | |
| --- | --- | --- | --- | --- | --- | --- | --- | --- | --- | --- |
|  |  | **80%** | **90%** | **95%** | **80%** | **90%** | **95%** | **80%** | **90%** | **95%** |
| <30 | Low | 10 | 13 | 15 | 15 | 18 | 21 | 19 | 22 | 26 |
|  | Intermediate | 6 | 8 | 9 | 9 | 11 | 13 | 12 | 14 | 16 |
|  | High | 5 | 7 | 8 | 8 | 9 | 11 | 10 | 12 | 13 |
| 30–33 | Low | 14 | 17 | 21 | 19 | 24 | 28 | 25 | 30 | 34 |
|  | Intermediate | 8 | 10 | 12 | 12 | 14 | 16 | 15 | 18 | 20 |
|  | High | 7 | 8 | 10 | 10 | 11 | 13 | 12 | 15 | 16 |
| 34–36 | Low | 18 | 23 | 28 | 26 | 32 | 37 | 33 | 40 | 46 |
|  | Intermediate | 10 | 13 | 16 | 15 | 18 | 21 | 19 | 23 | 26 |
|  | High | 8 | 10 | 12 | 12 | 14 | 17 | 15 | 18 | 21 |
| 37–40 | Low | 29 | 38 | 46 | 42 | 52 | 61 | 54 | 65 | 75 |
|  | Intermediate | 16 | 21 | 25 | 23 | 29 | 34 | 30 | 36 | 41 |
|  | High | 13 | 16 | 20 | 18 | 22 | 26 | 24 | 28 | 32 |
| >40 | Low | 68 | 89 | 108 | 98 | 121 | 143 | 126 | 153 | 177 |
|  | Intermediate | 37 | 47 | 57 | 52 | 65 | 76 | 68 | 82 | 94 |
|  | High | 28 | 36 | 44 | 40 | 49 | 58 | 52 | 62 | 72 |

Bold text is used for column headings only. Values are the minimum number of mature oocytes required to achieve the specified number of euploid blastocysts with the specified probability (80%, 90%, or 95%), estimated from the probabilistic framework described in the Materials and methods section. The base-case analysis reported in the main text corresponds to at least three euploid blastocysts at a probability of 90% or greater. No statistical comparisons were performed in this table. MAGENTA, an artificial-intelligence-derived oocyte quality score (0–10 scale) generated from a static image of the mature oocyte; tertile cut-offs were 3.70 and 8.40.
