# Supplementary material for: Are women cryopreserving enough oocytes for fertility preservation? A probabilistic model integrating AI-based oocyte morphology and age-dependent euploidy
Source: Hum Reprod Open. 2026 Oct 14;2026(4):hoag090. doi: 10.1093/hropen/hoag090 (PMC13690086; doi:10.1093/hropen/hoag090)
Supplement: Supplementary file 1 [file hoag090_supplementary_data.zip › Supplementary_Table_S1.docx]

**Supplementary Table S1.** Characteristics of the three datasets used for model development, external validation, and clinical application.

| **Dataset** | **Role in study** | **Period** | **Patients, n** | **Cycles, n** | **Oocytes / embryos, n** | **Maternal age, y (mean ± SD; range)** | **Outcome and PGT-A** |
| --- | --- | --- | --- | --- | --- | --- | --- |
| Autologous ICSI (blastulation subset) | Model development: blastulation | 2021–2024 | 1,020 | 1,625 | 13 782 MII oocytes | 36.9 ± 4.1; 21–50 | Blastulation 50.3%; no PGT-A in this subset |
| Autologous ICSI (euploidy subset) | Model development: euploidy | 2021–2024 | 857 | – | 3,325 biopsied blastocysts | 37.9 ± 3.5; 22–47 | Euploidy 36.3%; PGT-A by NGS |
| Ovum donation | External validation: blastulation | 2020–2024 | 271 | 333 | 1,948 MII oocytes | 26.8 ± 3.8; 18–36 | Blastulation 39.3%; no PGT-A |
| Fertility preservation | Real-world application | 2012–2024 | 1,100 | 1,647 | 6.6 ± 6.6 MII per cycle | 37.0 ± 3.8 | Cryopreserved MII per cycle; no PGT-A |

Bold text is used for column headings only. A dash indicates that the value is not applicable, because the euploidy subset was defined at the level of biopsied blastocysts rather than cycles. No statistical comparisons were performed in this table. PGT-A was performed only in the autologous ICSI euploidy subset; no PGT-A data were available for the ovum donation or fertility preservation datasets. The euploidy subset comprised biopsied blastocysts with a conclusive PGT-A result. MAGENTA tertile cut-offs (3.70 and 8.40) were applied uniformly to the autologous ICSI and ovum donation datasets, in which MAGENTA scores were available; MAGENTA scores were not available for the fertility preservation dataset. MAGENTA, an artificial-intelligence-derived oocyte quality score (0–10 scale) generated from a static image of the mature oocyte; MII, metaphase II; NGS, next-generation sequencing; PGT-A, preimplantation genetic testing for aneuploidy.
