# Supplementary material for: Are women cryopreserving enough oocytes for fertility preservation? A probabilistic model integrating AI-based oocyte morphology and age-dependent euploidy
Source: Hum Reprod Open. 2026 Oct 14;2026(4):hoag090. doi: 10.1093/hropen/hoag090 (PMC13690086; doi:10.1093/hropen/hoag090)
Supplement: Supplementary file 1 [file hoag090_supplementary_data.zip › Supplementary_Table_S3.docx]

**Supplementary Table S3.** Demographic and clinical characteristics of patients returning to use their cryopreserved oocytes in warming / ICSI cycles (n = 132).

| **Variable** | **Mean** | **SD** |
| --- | --- | --- |
| Maternal age, years | 38.0 | 3.8 |
| Body mass index, kg/m² | 24.6 | 3.7 |
| Aspirated follicles, n | 14.3 | 11.1 |
| Retrieved oocytes, n | 10.4 | 8.7 |
| Oocyte retrieval rate, % | 68.8 | 25.5 |
| Oocyte maturation rate, % | 70.0 | 25.8 |
| Survival rate, % | 91.6 | 36.8 |
| Injected oocytes, n | 7.6 | 4.2 |
| Fertilisation rate, % | 76.9 | 24.5 |
| Blastulation rate, % | 41.7 | 40.5 |
| Implantation rate, % | 41.5 | 42.2 |
| Cancellation rate, n/N (%) | 14/132 (10.6) | – |
| Clinical pregnancy rate, n/N (%) | 63/118 (53.4) | – |
| Miscarriage rate, n/N (%) | 5/63 (7.9) | – |

Bold text is used for column headings only. Continuous variables are expressed as mean and standard deviation; categorical outcomes are expressed as n/N (%), for which a standard deviation is not applicable (dash). Values refer to the 132 patients who returned to use their cryopreserved oocytes; maternal age is the age at oocyte warming. No statistical comparisons were performed in this table. ICSI, intracytoplasmic sperm injection.
